# Primary or metastatic hepatic carcinoma? A breast cancer patient after adjuvant chemotherapy and radiotherapy postoperatively with intrahepatic cholangiocarcinoma and review of the literature

**DOI:** 10.1186/s12957-016-0943-0

**Published:** 2016-07-15

**Authors:** Zhao-yun Liu, Ju-jie Sun, Ke-wen He, Pei-ying Zhuo, Zhi-yong Yu

**Affiliations:** School of Medicine and Life Sciences, University of Jinan-Shandong Academy of Medical Sciences, Jinan, Shandong People’s Republic of China; Department of Oncology, Shandong Academy of Medical Sciences, Shandong Cancer Hospital affiliated to Shandong University, 440 Ji-Yan Road, Jinan, 250117 Shandong Province People’s Republic of China

**Keywords:** Breast neoplasm, Liver metastases, Intrahepatic cholangiocarcinoma

## Abstract

**Background:**

The liver is a common site of metastases, followed by the bone and lung in breast cancer. The symptoms of hepatic metastases are similar to intrahepatic cholangiocarcinoma (ICC). ICC is rare, with an overall incidence rate of 0.95 cases per 100,000 adults. The incidence of ICC for patients with breast cancer is very uncommon. Breast cancer patient with ICC is easily misdiagnosed as hepatic metastases.

**Case presentation:**

We report a breast cancer patient postoperatively who was hospitalized because of having continuous irregular fever for 1 month. Antibiotics were given for 1 week without any significant effect. Her admission bloods revealed elevated levels of carcino-embryonic antigen. Magnetic resonance imaging diagnosis showed multiple liver metastases. We believed that the woman had hepatic metastases until biopsy guided by computed tomography. The liver biopsy pathology analysis considered the possibility of primary intrahepatic cholangiocarcinoma.

**Conclusions:**

Breast cancer patient with space-occupying lesions in the liver is easily considered to be progressed hepatic metastases. Image-guided biopsy is the best diagnostic method for breast cancer with liver mass to avoid misdiagnosis and classify the molecular subtypes to make appropriate treatment.

## Background

Intrahepatic cholangiocarcinoma (ICC) is a rare tumor, accounting for 3 % of all gastrointestinal malignancies worldwide [1–4]. With similar signs and symptoms to metastatic hepatic carcinoma including right abdominal pain, low-grade fever, nausea, anorexia, weight loss, and jaundice, ICC lacks typical clinical manifestations and is hard to differentiate from liver metastases using imaging modalities and tumor markers. For breast cancer, the liver metastatic is common [5, 6]. Approximately, in 10 % of the cases, distant metastases are already present at the time of diagnosis, and it is considered that more than 50 % of the patients with advanced breast cancer will develop hepatic metastasis at some point [7–9]. The main treatment to breast cancer with hepatic metastasis is systemic chemotherapy; however, curative resections are the only currently well-established option for ICC. Pathologic diagnosis is important to breast cancer patient with space-occupying lesions of the liver in order to avoid misdiagnosis and mistherapy. In the case presented here, we report a breast cancer patient postoperatively diagnosed with ICC other than hepatic metastases.

## Case presentation

**Fig. 1:**
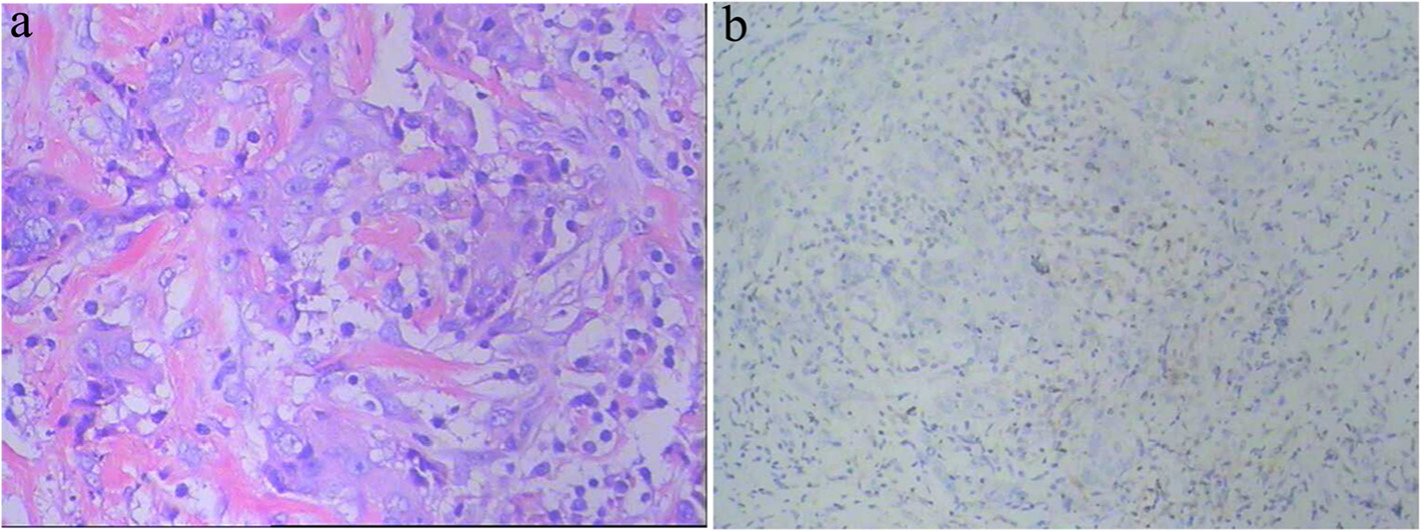
Photomicrographs of invasive breast carcinoma. **a** Light microscopy sections with hematoxylin and eosin staining demonstrating markedly atypical endothelial cells (HE, ×200). **b** The chief cells showed positive staining to CD10 (IHC, ×100)

A 48-year-old woman with “left breast cancer” was hospitalized in Shandong Cancer Hospital & Institute. This patient underwent modified radical mastectomy on July 18, 2014. Pathology diagnosis (Fig. 1): invasive ductal carcinoma, maximum diameter of the mass is 1.3 cm. Axillary lymph nodes involved level I 3/20, level II 0/20, and level III 0/1. The pathological staging was T_1_N_1_M_0_, stage IIA, which predicts the locally advanced disease. Immunohistochemistry results are the following: ER (−), PR (−), CerbB-2 positive (3+), and Ki-67+ (20 %). The patient refused targeted trastuzumab therapy because of personal economic situation. Chemotherapy regimen including AC (pirarubicin, 80 mg, d_1_; cyclophosphamide, 0.8 g, d_1_) sequential TP (docetaxel, 120 mg d_1_; cis-platinum, 60 mg d_1_, d_2_) was implemented after operation. Next, local radiotherapy was implemented on the left side of the chest wall and the left clavicle lymph drainage area, DT 50 Gy/25 times.

**Fig. 2:**
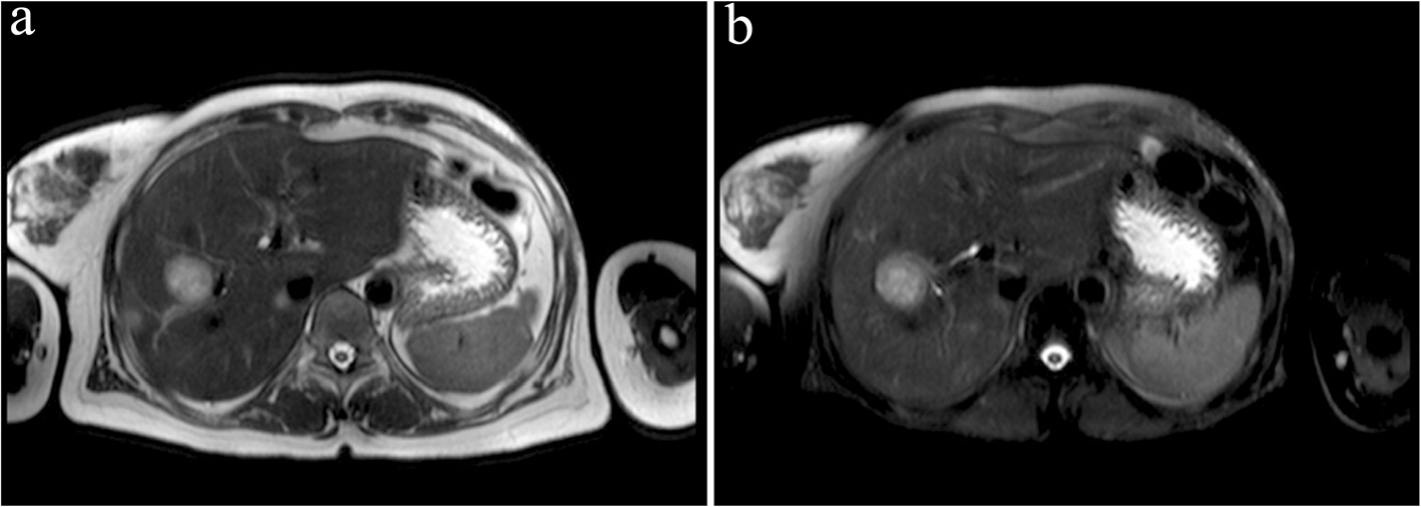
Liver magnetic resonance image (MRI) after the patient have continued irregular fever for 1 month

**Fig. 3:**
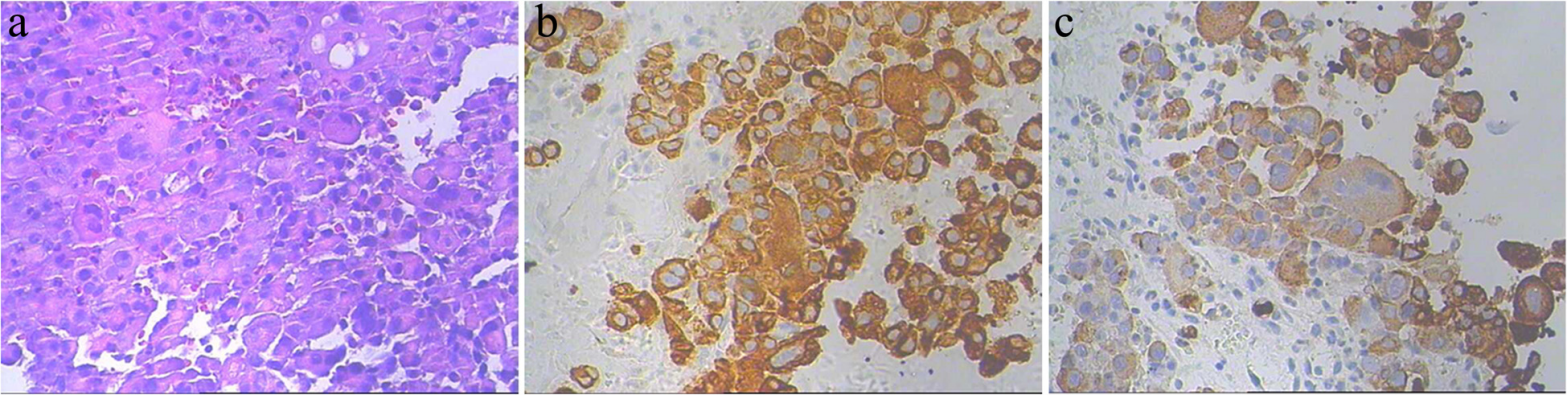
Photomicrographs of intrahepatic cholangiocarcinoma. **a** Eosin staining demonstrating markedly atypical cells (HE × 200). **b** CK7,cytoplasm + (IHC, ×200). **c** CK19,cytoplasm + (IHC, ×200)

Fourteen months later, this patient was again hospitalized on Sep.19, 2015, because of having continuous irregular fever for 1 month. Antibiotics were given for 1 week without any significant effect observed. We excluded the possibility of common infectious diseases. Computed tomography (CT) revealed low-density foci in the right liver lobe. The carcino-embryonic antigen (CEA) was 7.29 ng/mL, while the standard value is less than 3.4 ng/mL. We suspected the women had liver metastasis after breast cancer surgery, and the magnetic resonance imaging (MRI) abdomen diagnosis on Sep.20, 2015, was consistent with what we had suspected (Fig. 2). Neither cerebral MRI nor whole body bone scan revealed obvious abnormity or other distant metastases. However, on Sep.23, 2015, pathology analysis through row CT-guided liver biopsy demonstrated for poorly differentiated adenocarcinoma. Furthermore, the photomicrographs (Fig. 3) including eosin staining (HE), CK7, and CK19 accord with the characteristics of the low differentiation of intrahepatic bile duct carcinoma (ICC), which is the primary intrahepatic lesions.

### Discussion

Breast cancer, the most common malignant cancer in women worldwide, is a main cause of cancer death in females and the second in the general population just after lung cancer. Liver metastases have a 5-year survival rate of 0 to 12 %, which is the poorest prognosis of all types of breast cancer metastases [10]. Metastases seed the liver via systemic circulation with a microscopic dissemination and may be present in multiple sites [11–13]. Considering this patient with lymph node metastasis and positive CerbB-2 expression, she was suspected of getting liver metastases first, and the MRI imaging approved this hypothesis. Symptoms such as abdominal fullness, back pain, fatigue, nausea, anorexia, low-grade fever, and weight loss usually occur during the progression of liver metastasis. In this case, we hypothesize that the fever of the woman may result from tumor tissue necrosis, infection, and tumor metabolites. The vast majority of patients with liver metastasis from breast cancer also have metastasis in other parts of their bodies. Thus, systemic treatment served as a better choice. Due to the limitation of operation range, surgical practice in patients with liver metastasis from breast cancer is still under debate. The majority of clinicians do not support surgery with the role of surgery being strictly limited for palliation of metastatic complications or locoregional relapse, while others supported this method for its cost-effectiveness with superior 5-year survival for selected patients with isolated liver metastases and in those with well-controlled minimal extrahepatic disease [14, 15].

ICC is one of the primary malignant liver tumors with a prevalence of 5–20 % in hepatic carcinoma cases [16]. Although patients with ICC exhibited hepatic region pain, weight loss, or few of irregular fever, a considerable number of patients are asymptomatic [17, 18]. The patient in our report had fever as the first symptom is easily misdiagnosed. The nonspecific tumor markers CEA can be found elevated both in ICC and in liver metastasis. It is not helpful for us to do a differential diagnosis with this woman who only had CEA elevated [17]. Imaging features of ICC are space-occupying lesions in the liver [19], which we are easily confused with liver metastases. Due to the challenges in their detection and treatment, ICC is usually already progressed invasively and transfers to the lymphatic system during its early stage [20, 21]. Though the resectability and curability remain low compared to other hepatobiliary tumors, surgical resection is the preferred treatment for the ICC [22].

This breast cancer patient 1 year after surgery has continuous irregular fever for 1 month, and 1-week intravenous antibiotics were of no use. With the MRI imaging findings and elevated levels of CEA, as well as the unstandardized treatment of HER2 target, we easily believed that this breast cancer patient had hepatic metastases. But the liver biopsy pathology analysis by CT-guided biopsy led us to consider the possibility of primary ICC.

## Conclusions

This breast cancer patient postoperatively was diagnosed to hepatic metastases with the clinical symptoms and imaging diagnosis until the biopsy results were reported to primary ICC. For breast cancer patients with liver space-occupying lesions, we should not treat them imprudently to the liver metastasis of breast cancer. Instead, we should make careful judgment bases on the biopsy and pathology diagnosis, providing the basis for subsequent treatment. Imaging-guided biopsy should be chosen to breast cancer patient with space-occupying lesions of the liver in order to avoid misdiagnosis and classify the molecular subtypes to make appropriate treatment.

## Abbreviations

CEA, carcino-embryonic antigen; CT, computed tomography; ICC, intrahepatic cholangiocarcinoma; MRI, magnetic resonance imaging
